# Knowledge and perceptions of Alzheimer’s disease in three ethnic groups of younger adults in the United Kingdom

**DOI:** 10.1186/s12889-021-11231-8

**Published:** 2021-06-12

**Authors:** Aysegul Humeyra Kafadar, Christine Barrett, Kei Long Cheung

**Affiliations:** Department of Health Sciences, Brunel University London, Uxbridge, UB8 3PH UK

**Keywords:** Dementia, Alzheimer’s disease, Ethnicity, ADKS, Knowledge, United Kingdom

## Abstract

**Background:**

Alzheimer’s disease (AD) is a global public health problem with an ageing population. Knowledge is essential to promote early awareness, diagnosis and treatment of AD symptoms. AD knowledge is influenced by many cultural factors including cultural beliefs, attitudes and language barriers. This study aims: (1) to define AD knowledge level and perceptions amongst adults between 18 and 49 years of age in the UK; (2) to compare knowledge and perceptions of AD among three main ethnic groups (Asian, Blacks, and Whites); and (3) to assess potential associations of age, gender, education level, affinity with older people (65 or over), family history and caregiving history with AD knowledge.

**Methods:**

Data was collected from 186 participants as a convenience sample of younger adults of three different ethnicities (16.1% Asian, 16.7% Black, 67.2% White), living in the UK, recruited via an online research platform. The majority of the participants were in the 18–34 years age group (87.6%). Demographic characteristics of participants and AD knowledge correlation were assessed by the 30-item Alzheimer’s Disease Knowledge Scale (ADKS), comprising 7 content domains. ANOVA/ANCOVA were used to assess differences in AD knowledge by ethnicity, gender, education level, age and affinity with dementia and Alzheimer’s patients.

**Results:**

For AD general knowledge across all respondents only 45.0% answers were correct. No significant differences were found for the total ADKS score between ethnicities in this younger age group, who did not differ in education level. However, there were significant knowledge differences for the ADKS symptom domain score even after controlling for other demographics variables such as gender, education level (*p* = 0.005). White respondents were more likely to know about AD symptoms than their Black counterparts (*p =* 0.026).

**Conclusion:**

The study’s findings suggest that the AD knowledge level is not adequate for all ethnic groups. Meanwhile, significant differences were observed in symptoms, between ethnic groups, and therefore, differ in their needs regards health communication. The study contributes to an understanding of ethnicity differences in AD knowledge amongst adults from 18 to 49 years of age in the UK and may also provide input into an intervention plan for different ethnicities’ information needs.

## Background

Dementia has become a priority global public health problem in the context of the ageing populations [1], affecting approximately 44 million individuals with dementia worldwide. Approximately 850,000 individuals have dementia in the UK [2]. It is estimated this number will reach 2 million by 2050. In 2019, dementia resulted in a total cost to the UK economy of £34.7 billion [3]. Alzheimer’s Disease (AD) is the most common type of dementia and is diagnosed in almost 75% of cases in the UK [4, 5]. AD is a progressive and irreversible neurodegenerative disease. It does not only lead to loss of memory but also causes deterioration in other fields of mental function and in activities of daily living, accompanied by changes in personality and behaviour [6–9]. Therefore, AD results in a complex economic burden for patients and their caregivers due to long-lasting disabilities, increased dependency, costs of direct medical and non-medical care such as therapies, resource utilisation, emergent behavioural problems, and loss of functionality [10–12].

Protecting individuals from AD risk factors is of primary importance [13]. The risk of having AD can be decreased when engaging in certain lifestyle behaviours, e.g. physical activity, regular sleep, abstinence from tobacco smoking, and socialisation [6, 14, 15]. That is because the AD risk increases with several preventable factors such as obesity, hypertension, type-2 diabetes, tobacco use, and depression [6, 16–18]. Such risk factors are often already apparent in a younger population decades before typical age of onset of AD [19, 20].

Previous studies confirm these results and demonstrate that awareness was most lacking for initiative dimensions and emotional blunting for AD patients [21, 22]. Socio-cognitive theories also showed that awareness is a prerequisite to changing behaviours [23], and deficiency of awareness causes different interpretation of public health communication messages for behaviour change [23–25]. To increase awareness of individuals, a person’s knowledge, risk perception, behaviour cognizance, and perceived cues should be increased or changed [23–25]. Since these factors may be affected by ethnicity, firstly differences in perception and information needs of ethnic groups should be determined.

Ethnic groups may differ in knowledge and perceptions with regards to AD due to differences in socio-economic variables (e.g. educational level, income level, and having a family member with AD) as well as cultural drivers [26, 27]. For example, Anglo older adults have higher levels of AD knowledge than their Latino counterparts in the USA, which was explained by education level [26]. Moreover, in the same study it has been found that Latino older adults had more information related to AD than their Asian counterparts, which was explained by years of speaking English [26]. The knowledge and perception differences may impact on noticing signs of disease, help-seeking behaviours and health-related life quality [26]. Moreover, potential differences in perceptions between ethnic groups may affect AD and dementia knowledge [17, 28, 29]. For example, a systematic review showed that dementia and AD have been perceived as a normal situation when becoming older among racial and ethnic minorities [30, 31]. Likewise, in several previous studies, Black people, Chinese and Hispanics in the USA were more likely to expect that AD is a normal part of ageing than their white counterparts [17, 28].

Decreased knowledge levels may lead to stigmatisation in some minority ethnic groups. The stigma can impact individuals’ beliefs and attitudes regarding AD, which may link to help-seeking behaviours [27, 32, 33]. Research investigating knowledge of dementia and AD among individuals from ethnic minorities is limited outside the USA, with the exception of several studies from the UK [27, 34, 35].

Studies have shown that there are numerous racial differences in the experiences of family caregivers’ interventions for dementia and AD amongst different ethnic groups, in African American, Latino, and Chinese Americans in the US [36]. These ethnic differences have shown up in multiple areas such as caregiving appraisal, life satisfaction, psychosocial health, coping, spirituality, social support, self-efficacy, filial responsibility, physical functioning, familism, opinions towards older people and use of health care and formal services [36, 37]. In a previous study in the US, African Americans notably indicated that physicians disrespect the caregivers’ regarding memory loss concerns; Chinese participants, on the other hand, remarked on stigmatisation of individuals with AD. Moreover, Latinos reported their fears that acculturation would end family home care in the USA (mean age 53) [38].

In the UK, the Manchester study compared dementia knowledge between Indian and Caucasian older people in 2006. It was found that Indian older people had inadequate knowledge about dementia by of their relative absence in the local dementia treatment clinics. Additionally, many Indian participants stated that they probably did not recognize dementia because they considered many of its symptoms to be a normal aging [35]. In another UK study, although South Asian and black minority ethnic groups were found to have dementia four to five years earlier than White British population, they were more likely to have late diagnosis because of language difficulties, under-representation in memory health services, and lack of encouragement for help-seeking [34]. As a result, previous studies indicated that ethnic minorities may have different knowledge and perceptions regarding dementia and AD [17, 26–31]. Hence, there is a need to understand these inequalities, to see if dementia and AD prevention initiatives should be tailored by ethnic group and to support their diagnosis across all ethnic groups as early as possible. The lack of research on the differences in knowledge and risk perception between ethnic minorities may result in the development of suboptimal health communication. Hence, it is important to understand the differences in level of knowledge, so that health communication strategies can be optimised for the UK, and understanding knowledge differences between groups will help inform the design of targeted health communication interventions [23–25, 34]. The goal of this study is therefore to understand the level of knowledge regarding AD in the UK and explore potential differences between major ethnic groups.

Literature shows that AD knowledge and perception status have been affected by many demographic factors such as age, gender, marital status, education level, language differences, as well as ethnicity [17, 26–28, 30, 31, 39–41]. In Bond et al’s study of older adults (ranged in age from 50 to 92), lack of awareness and knowledge of symptoms, treatment and supportive care options have been defined as barriers to improving the availability and development of treatments and support organisations for individuals with AD [42]. It was found that there was better knowledge regarding problems concerning diagnosis, treatment, and management of AD amongst the younger, better educated and university professionals than older and less educated people in the Brazilian population [43]. The unemployed or retired individuals were shown to have poor perceptions and lower levels of knowledge about dementia and AD than employed people or students, in China [39]. Another study indicated that older Chinese women had more information on AD than men when educational differences were inspected [41]. Furthermore, it was found that being male, old age, and having lower education levels were correlated with AD information paucity amongst Chinese participants in the US [37]. However, in other literature, there was evidence that background characteristics such as age, gender, and education that these may not have a high significant impact on AD awareness [27, 44]. Thus, the impact of demographic features on AD knowledge and perceptions is unclear, indicating the need to explore how demographic differences relate to AD and dementia knowledge.

As mentioned above, previous studies have tended to research dementia or AD knowledge amongst older population (50 years or over) [17, 26, 27, 39, 41, 42, 45] because the diseases commonly affect older individuals. However, in several studies of the general population in the UK (average of age from 18 to 43), AD knowledge has been shown to be inadequate in younger adults [44, 46]. Importantly, AD knowledge related to AD risk factors and the course of the disease were found to be lower than AD life impact, assessment and diagnosis, treatment and management, and symptoms knowledge amongst younger adults [44]. Lastly, knowledge of AD treatment has been determined as insufficient amongst 18 years or over years individuals [46]. To the best of our knowledge, there is a paucity of studies which specifically research AD knowledge between ethnicities for those younger than 50 years in the UK. The situation creates a knowledge gap for young adults’ AD knowledge and perceptions in different ethnic groups in the UK. Therefore, in this study, we focused on analysing AD knowledge amongst adults from 18 to 49 years of age rather than adults 50 years and over. To determine public health interventions which increase AD knowledge level and change perceptions of AD for population protection from AD, understanding the knowledge status among younger adults about risk factors, symptoms, diagnosis, or assessment criteria of AD is more important than in older adults.

This leads to the aim of this study, which is threefold: (1) to define AD knowledge level and perceptions amongst adults between 18 and 49 years of age in the UK; (2) to compare knowledge and perceptions of AD among three main ethnic groups (Asian, Blacks, and Whites); and (3) to assess potential associations of age, gender, education level, affinity with older people (65 or over), family history and caregiving history with AD knowledge.

## Methods

The cross-sectional study explored self-reported AD knowledge and perceptions amongst adults from 18 to 49 years of age in the UK. An online survey was designed for primary data collection.

### Participants and recruitments

The study was originally designed to focus on adults in the UK and compared three ethnic groups: (1) White people, (2) Black people, and (3) Asian people. The inclusion criteria were, therefore: (1) aged 18 years and over; and (2) resident in the UK (who were either born in the UK or had migrated to the UK). Due to a very low response from those 50 and over, and to align with a recognised knowledge gap in younger adults, the inclusion criteria for analysis was restricted to those aged 18 to 49 years.

In order to detect a one-point knowledge difference for AD between three ethnic groups on our main measure (Alzheimer’s Disease Knowledge Scale (ADKS) total score) with 80% power at a 5% significance level (α = 0.05), power calculation showed that at least 158 participants were needed in this study with at least 30 participants per ethnic group.

The convenience sampling was conducted via an online research platform from Prolific Academic Ltd. [47] at a time when restrictions due to the COVID-19 pandemic limited other study designs and recruitment options. The pre-screening questions from Prolific identified 87,336 potential participants who were currently residing in the UK, were at least 18 years old, and were from these three different ethnic groups, White, Asian, or Black. Participants were recruited to the study on a first-come, first-served basis following receipt of an email from Prolific to a random subset of all potentially eligible participants. To ensure sufficient numbers per ethnic group, the sample was enriched for those of Black and Asian ethnicity by additional recruitment. At the end of recruitment process, we recruited 186 participants aged 18 to 49 years old, out of 190 who responded.

### Procedure

In June 2020, data were collected using an online survey hosted by Bristol Online Survey. Before the survey, all participants were informed about the study’s purpose, that participation was anonymous, voluntary, that they were free to withdraw their data without giving a reason until the point at which they submit their answers, and the required time commitment. When participants logged into the survey portal through Prolific, they first read the Participant Information Sheet, then were presented with a questionnaire. Participants who provided electronic written consent (though initial survey questions) and completed the survey received an incentive of £1.40 for the approximately 10 min time requirement to complete the survey.

### Questionnaire

The Bristol Online Surveys application was used to create an online survey with closed-ended questions.

#### Demographic data

Participants’ demographic characteristics were collected to show whether an association between them and Alzheimer’s knowledge score existed. Gender was recorded as male, female, other, or prefer not to answer. Age was originally coded as an ordinal variable with four age groups (18–34 years, 35–49 years, 50–64 years, 65 years old and more) with the analysis dataset reduced to the 18–34- and 35–49-years groups. Education level information was categorised as an ordinal variable: no schooling completed, nursery school to 8th grade, high school graduate, diploma or the equivalent (for example, GED, GCSE and ‘A’ level), bachelor’s degree, master’s degree, or doctorate degree. A dichotomised education variable of graduate and non-graduate was also derived. Ethnicity was categorised into a three-group variable (1 = White; 2 = Black; 3 = Asian).

The questionnaire also included three questions related to affinity with dementia and Alzheimer’s patients, first “Are there any people with dementia and/or Alzheimer’s Disease who are close to you?” (0 = Yes, 1 = No); second “Have you ever lived with people older than 65 years of age?” (0 = No, 1 = I have lived with older people who do not have dementia and/or Alzheimer’s disease, 2 = I have lived with older people with dementia and/or Alzheimer’s disease). Lastly, “Have you ever interacted with people with dementia and/or Alzheimer’s disease?” (0 = No, 1 = For several times, 2 = From several weeks to about a year, 3 = For more than a few years).

#### Alzheimer’s disease knowledge scale (ADKS)

AD knowledge was assessed with a 30-item ADKS scale, which is a commonly used scale designed to measure AD knowledge. It was found that the test-retest reliability coefficient was 0.81, *p* < 0.001, revealing adequate test-retest reliability, coefficient alpha was 0.71 (internal consistency), and split-half reliability was 0.55, p < 0.001, while content, concurrent, predictive, and convergent validity of ADKS was confirmed [48]. The primary outcome of concern was the ADKS score. The ADKS consists of 30 true-false questions covering the following 7 content domains: risk factors (6 items assessing the knowledge about AD risk factors, e.g. high blood pressure, high cholesterol, genetics, ageing), symptoms (4 items assessing the knowledge about symptoms of AD, e.g. tremor and shaking are not symptoms for AD, troubles for handling money or paying bills, and remembering past events better than things that happened in recent days are symptoms of AD), course of the disease (4 items assessing the knowledge about AD process, e.g. the average life expectancy is 6 to 12 years after symptoms of AD appear, people have not recovered from AD, a person with AD needs 24­hour supervision), assessment and diagnosis (4 items assessing the knowledge of AD assessment and diagnosis, e.g. AD is one type of dementia, memory problem and confused thinking which are suddenly appear it is not likely due to AD), treatment and management (4 items assessing the knowledge of AD treatment and management process, e.g. poor nutrition can make the symptoms of AD worse, using reminder notes is a crutch that cannot contribute to decline AD symptoms), life impact (3 items assessing the knowledge about AD effects on life, e.g. people with AD are particularly prone to depression, driving car is not safe for people with AD), and caregiving (5 items assessing the knowledge of AD care, e.g. people with AD do best with simple, instructions given one step at a time, if a person with AD becomes alert and agitated at night a good strategy is to try to make sure that the person gets plenty of physical activity during the day, and people with AD can be capable of making informed decisions about their own care) for evaluation of knowledge of AD to determine knowledge status of the age group (younger than 50 years of age) in this study [48, 49]. The questionnaire also includes a “do not know” section to obtain more reliable data [50]. The resulting score is the number of questions answered correctly, giving a total score with a range of 0–30, where a higher total score indicates that the participant has better knowledge [49, 51, 52].

### Statistical analysis

Data contained no missing values. Analyses were conducted with IBM SPSS Statistics, version 26. A Shapiro-Wilk’s test (*p* > 0.05) [53] and a visual inspection of histograms, normal Q-Q plots and box plots showed that the ADKS total score was approximately normally distributed across all independent variables (age, gender, ethnicity, education level, living with older people (65 or over), familiarity people with AD and/or dementia, and interaction with people with AD and/or dementia) [53–55]. Descriptive statistics were used to determine sample characteristics of variables, ADKS items, the ADKS domain scores, as well as percentage of correct answers, total and domain ADKS scores by ethnicity.

To compare knowledge and perceptions of AD among three ethnic groups (Asian, Black, and White), univariate analyses were conducted using ANOVA and MANOVA to investigate whether there were differences between the three ethnic groups in terms of total or domain ADKS scores. ANCOVA was used to assess ethnicity association with ADKS when controlling for demographic variables as covariates. Differences between demographic variables (such as gender and education), and ethnic groups were assessed with Pearson’s χ^2^ test.

In order to assess the association between other demographic variables and AD knowledge, we conducted ANOVA tests with age, gender, educational level, affinity with AD, living with 65 or over people, and whether they interacted with people with dementia or AD as independent variables and ADKS total or domain score as the dependent variable. Finally, MANOVA tests were used for multivariate analysis across the domains for each independent variable, with a MANCOVA test for ethnicity controlled for covariates.

## Results

### Demographic characteristics

Amongst the 186 participants in these analyses, 67.2% were White, and 57.0% were male. The majority of the participants (87.6%) were in the 18–34 years age group, and 54.8% of respondents were non-graduates. Approximately 74.2% of participants stated that they do not have a relative with dementia and/or AD, and 46.8% of respondents stated that they had not interacted with people with dementia and/or AD, while only 13.4% of respondents reported that they lived with older people with dementia and/or AD. No significant differences were found in demographic variables, including education, between ethnic groups. The demographic and ethnic groups of the participants are summarised in Table 1.

**Table 1:** Descriptive Characteristics of Participants (*n* = 186), by Race/Ethnicity

Characteristics	White (*n* = 125; 67.2%)	Black (*n* = 31; 16.7%)	Asian (*n* = 30; 16.1%)	Total (n = 186; 100%)
**Age Groups (*n*** **= 186)**				
18–34 years	111 (88.8%)	26 (83.9%)	26 (86.7%)	163 (87.6%)
35–49 years	14 (11.2%)	5 (16.1%)	4 (13.3%)	23 (12.4%)
**Sex (n = 186)**				
Female	53 (42.4%)	12 (38.7%)	15 (50.0%)	80 (43.0%)
Male	72 (57.6%)	19 (61.3%)	15 (50.0%)	106 (57.0%)
**Education level (n = 186)**				
Non-Graduate	69 (55.2%)	16 (51.6%)	17 (56.7%)	102 (54.8%)
No schooling completed	3 (2.4%)	0 (0%)	0 (0%)	3 (1.6%)
Nursery school to 8th grade	2 (1.6%)	1 (3.2%)	0 (0%)	3 (1.6%)
High school graduate, diploma or the equivalent (for example, GED, GCSE and ‘A’ level)	64 (51.9%)	15 (48.4%)	17 (56.7%)	96 (51.6%)
Graduate	56 (44.8%)	15 (48.4%)	13 (43.3%)	84 (45.2%)
Bachelor’s degree	41 (32.8%)	10 (32.3%)	10 (33.3%)	61 (32.8%)
Master’s degree	13 (10.4%)	4 (12.9%)	2 (6.7%)	19 (10.2%)
Doctorate degree	2 (1.6%)	1 (3.2%)	1 (3.3%)	4 (2.2%)
**Familiarity with AD/dementia (n = 186)**				
Yes	34 (27.2%)	8 (25.8%)	6 (20.0)	48 (25.8%)
No	91 (72.8%)	23 (74.2%)	24 (80.0%)	138 (74.2%)
**Living with 65 or over years old (n = 186)**				
No	53 (42.4%)	17 (54.8%)	10 (33.3%)	80 (43%)
Living with 65 or over people without dementia/AD	53 (42.4%)	12 (38.7%)	16 (53.3%)	81 (43.5%)
Living with 65 or over people with dementia/AD	19 (15.2%)	2 (6.5%)	4 (13.3%)	25 (13.4%)
**Interacted with people with dementia/AD (n = 186)**				
No	57 (45.6%)	16 (51.6%)	14 (46.7%)	87 (46.8%)
For several times	44 (35.2%)	6 (19.4%)	10 (33.3%)	60 (32.3%)
From several weeks to about a year	8 (6.4%)	1 (3.2%)	3 (10.0%)	12 (6.5%)
For more than a few years	16 (12.8%)	8 (25.8%)	3 (10.0%)	27 (14.5%)

### Knowledge and perceptions of dementia and Alzheimer’s disease in younger adults in the UK

In order to define AD knowledge level and perceptions amongst adults between 18 and 49 years of age of different ethnicities in the UK, descriptive analyses were conducted. In total, 45.0% of answers were correct; the total mean score of the ADKS was 13.5 of 30 (SD **±** =5.4 in the study sample (Table 3). The majority of participants (82.8%, *n* = 154) did not respond correctly to the statement that ‘It has been scientifically proven that mental exercise can prevent a person from getting Alzheimer’s disease’. Most participants (79.0%, *n* = 147) responded correctly that ‘People with Alzheimer’s disease do best with simple instructions given one step at a time’. The percentage of participants who correctly answered each ADKS item is illustrated in Table 2. Items with the poorest responses, included those related to AD’s risk factors (35.3% correct answers, Mean = 2.1, SD **± =**1.3 in 6 items). Conversely, items with the highest correct responses included those related to life impact (50.8% correct answers), and AD’s assessment and diagnosis (50.1% correct answers). The percentage correct answers, mean, and SD **±** for ADKS total and domains are summarised in Table 3.

**Table 2:** Percentage of Participants Who Correctly Answered Each ADKS Item (*n* = 186), by Ethnicity

ADKS item	Domain	Answer	White (*n* = 125; 67.2%)	Black (*n* = 31; 16.7%)	Asian (*n* = 30; 16.1%)	Total (n = 186; 100%)
1. People with Alzheimer’s disease are particularly prone to depression.	Life Impact	True	49 (39.2%)	18 (58.1%)	14 (46.7%)	81 (43.5%)
2. It has been scientifically proven that mental exercise can prevent a person from getting Alzheimer’s disease.	Risk Factors	False	25 (20.0%)	4 (12.9%)	3 (10.0%)	32 (17.2%)
3. After symptoms of Alzheimer’s disease appear, the average life expectancy is 6 to 12 years.	Course of the Disease	True	43 (34.4%)	8 (25.8%)	6 (20.0%)	57 (30.6%)
4. When a person with Alzheimer’s disease becomes agitated, a medical examination might reveal other health problems that caused the agitation.	Assessment and Diagnosis	True	55 (44.0%)	18 (58.1%)	10 (33.3%)	83 (44.6%)
5. People with Alzheimer’s disease do best with simple, instructions giving one step at a time.	Caregiving	True	99 (79.2%)	23 (74.2%)	25 (83.3%)	147 (79.0%)
6. When people with Alzheimer’s disease begin to have difficulty taking care of themselves, caregivers should take over right away.	Caregiving	False	35 (28.0%)	7 (22.6%)	9 (30.0%)	51 (27.4%)
7. If a person with Alzheimer’s disease becomes alert and agitated at night, a good strategy is to try to make sure that the person gets plenty of physical activity during the day.	Caregiving	True	54 (43.2%)	12 (38.7%)	14 (46.7%)	80 (43.0%)
8. In rare cases, people have recovered from Alzheimer’s disease.	Course of the Disease	False	55 (44.0%)	10 (32.3%)	10 (33.3%)	75 (40.3%)
9. People whose Alzheimer’s disease is not yet severe can benefit from psychotherapy for depression and anxiety.	Treatment and Management	True	62 (49.6%)	19 (61.3%)	15 (50.0%)	96 (51.6%)
10. If trouble with memory and confused thinking appears suddenly, it is likely due to Alzheimer’s disease.	Assessment and Diagnosis	False	61 (48.8%)	21 (67.7%)	10 (33.3%)	92 (49.5%)
11. Most people with Alzheimer’s disease live in nursing homes.	Life Impact	False	51 (40.8%)	10 (32.3%)	13 (43.3%)	74 (39.8%)
12. Poor nutrition can make the symptoms of Alzheimer’s disease worse.	Treatment and Management	True	57 (45.6%)	16 (51.6%)	8 (26.7%)	81 (43.5%)
13. People in their 30s can have Alzheimer’s disease.	Risk Factors	True	62 (49.6%)	16 (51.6%)	15 (50.0%)	93 (50.0%)
14. A person with Alzheimer’s disease becomes increasingly likely to fall down as the disease gets worse.	Course of the Disease	True	67 (53.6%)	15 (48.4%)	13 (43.3%)	95 (51.1%)
15. When people with Alzheimer’s disease repeat the same question or story several times, it is helpful to remind them that they are repeating themselves.	Caregiving	False	67 (53.6%)	15 (48.4%)	9 (30.0%)	91 (48.9%)
16. Once people have Alzheimer’s disease, they are no longer capable of making informed decisions about their own care.	Caregiving	False	49 (39.2%)	11 (35.5%)	12 (40.0%)	72 (38.7%)
17. Eventually, a person with Alzheimer’s disease will need 24­hour supervision	Course of the Disease	True	83 (66.4%)	15 (48.4%)	18 (60.0%)	116 (62.4%)
18. Having high cholesterol may increase a person’s risk of developing Alzheimer’s disease.	Risk Factors	True	26 (20.8%)	4 (12.9%)	6 (20.0%)	36 (19.4%)
19. Tremor or shaking of the hands or arms is a common symptom in people with Alzheimer’s disease.	Symptoms	False	46 (36.8%)	6 (19.4%)	6 (20.0%)	58 (31.2%)
20. Symptoms of severe depression can be mistaken for symptoms of Alzheimer’s disease.	Assessment and Diagnosis	True	49 (39.2%)	11 (35.5%)	7 (23.3%)	67 (36.0%)
21. Alzheimer’s disease is one type of dementia.	Assessment and Diagnosis	True	86 (68.8%)	23 (74.2%)	22 (73.3%)	131 (70.4%)
22. Trouble handling money or paying bills is a common early symptom of Alzheimer’s disease.	Symptoms	True	52 (41.6%)	11 (35.5%)	9 (30.0%)	72 (38.7%)
23. One symptom that can occur with Alzheimer’s disease is believing that other people are stealing one’s things.	Symptoms	True	71 (56.8%)	17 (54.8%)	10.(33.3%)	98(52.7%)
24. When a person has Alzheimer’s disease, using reminder notes is a crutch that can contribute to decline.	Treatment and Management	False	43 (34.4%)	10 (32.3%)	7 (23.3%)	60 (32.3%)
25. Prescription drugs that prevent Alzheimer’s disease are available.	Risk Factors	False	56 (44.8%)	14 (45.2%)	9 (30.0%)	79 (42.5%)
26. Having high blood pressure may increase a person’s risk of developing Alzheimer’s disease.	Risk Factors	True	22 (17.6%)	4 (12.9%)	10 (33.3%)	36 (19.4%)
27. Genes can only partially account for the development of Alzheimer’s disease.	Risk Factors	True	84 (67.2%)	20 (64.5%)	14 (46.7%)	118 (63.4%)
28. It is safe for people with Alzheimer’s disease to drive, as long as they have a companion in the car at all times.	Life Impacts	False	84 (67.2%)	26 (83.9%)	19 (63.3%)	129 (69.4%)
29. Alzheimer’s disease cannot be cured.	Treatment and Management	True	80 (64.0%)	16 (51.6%)	19 (63.3%)	115 (61.8%)
30. Most people with Alzheimer’s disease remember recent events better than things that happened in the past.	Symptoms	False	73 (58.4%)	11 (36.7%)	17 (56.7%)	101 (54.6%)
**Total Mean ADKS Score**			**14.0 (SD ± 5.6)**	**13.3 (SD ± 4.7)**	**12.0 (SD ± 5.6)**	**13.5 (SD ± 5.5)**

**Table 3:** ADKS Total and Content Domains Scores for Ethnic Groups (n = 186)

Content Domain	#items	Mean SD±	% Correct	White (n = 125) Mean SD±	Black (n = 31) Mean SD±	Asian (n = 30) Mean SD±	F value	*P*-value	Significant difference^a^
ADKS	30	13.5 (5.4)	45.0%	14.0 (5.5)	13.3 (4.7)	12.0 (5.6)	1.675	0.190	
Risk Factor	6	2.1 (1.3)	35.3%	2.2 (1.4)	2.0 (1.4)	1.9 (1.2)	0.742	0.478	
Symptoms	4	1.8 (1.1)	44.2%	1.9 (1.1)	1.4 (1.1)	1.4 (0.9)	4.285	0.015*	White*> Asian
Course of the Disease	4	1.8 (1.2)	46.1%	2.0 (1.2)	1.5 (1.3)	1.6 (1.2)	2.502	0.085	
Assessment and Diagnosis	4	2.0 (1.1)	50.1%	2.0 (1.1)	2.4 (1.0)	1.6 (1.1)	3.286	0.040*	Black*> Asian
Treatment and Management	4	1.9 (1.1)	47.3%	1.9 (1.1)	2.0 (1.1)	1.6 (1.1)	1.012	0.365	
Life Impact	3	1.5 (0.9)	50.8%	1.5 (0.9)	1.7 (0.9)	1.5 (1.0)	1.072	0.344	
Caregiving	5	2.4 (1.3)	47.4%	2.4 (1.3)	2.2 (1.4)	2.3 (1.2)	0.496	0.610	

^a^Significant differences on interval variables were defined using univariate analysis of variance. Post-hoc analysis using the Games-Howell procedure was conducted. * *p* < 0.05, < > indicates direction of differences

### Associations between ethnic groups and ADKS total and domain scores

Univariate ANOVA was used to compare ethnic group differences in knowledge of AD. As shown in Table 3, there was no statistically significant ethnic group difference in total ADKS score. However, White participants had numerically higher scores for total ADKS score than Black and Asian participants (14.0, 13.3, and 12.0, respectively). The ADKS domain scores were compared between each ethnic groups. Significant differences were observed in symptoms, and assessment and diagnosis domains (*p < 0.05).* While 44.2% of total respondents gave a correct *answer* to symptom items, significant ethnic group differences in knowledge of AD’s symptom domain were found *F* (2,183) = 4.285, *p* = 0.015. Post hoc comparison using the Games-Howell procedure, as appropriate for unequal sample group sizes, indicated that White participants scored higher than Asian participants on the symptom domain (*p = 0.025)*. In addition, although 50.8% of participants gave correct answers to the assessment and diagnosis content domain, significant ethnic group differences in knowledge of AD’s assessment and diagnosis domain were found, with *F* (2,183) *=* 3.286, *p* = 0.040. Post hoc comparison using the Games-Howell procedure indicated that Black respondents scored higher than Asian individuals (*p* = 0.027). On the remaining domains, there were no significant knowledge differences found amongst these three ethnic groups. Lastly, according to the ANCOVA test, there was also no significant effect of ethnicity on total ADKS score after controlling for the effects of gender, age, education level, familiarity with dementia, living with those over 65, and interacting with those with dementia, *F* (2,177) = 2.017, *p* = 0.136, partial η^2^ = 0.022. At a domain level, significant differences were found for symptom score after controlling ethnicity for covariates (*F* (2,177) = 5.363, *p* = 0.005, partial η^2^ = 0.057), with White > Black (*p* = 0.026), but not for the assessment and diagnosis score.

### Associations between other demographic characteristics and ADKS score

In order to explore whether demographic characteristics were associated with AD knowledge perceptions, univariate analyses were conducted using ANOVA for total ADKS score and within MANOVA for multiple dependent variables for the domain scores. According to these analyses, there was no statistically significant association between ADKS total or domain scores and age or education. However, other demographic independent variables were found to be significantly associated with different ADKS domains but not total score. Gender was significantly associated with the course of the disease knowledge domain, (*F*(1,184) = 6.236, *p* = 0.013), familiarity with people with AD/or dementia was significantly associated with symptom knowledge (*F*(1,184) = 11.195, *p* = 0.001) and living with 65 years old or over was significantly associated with caregiving knowledge score (*F*(2,183) = 7.332, *p* = 0.001), the interactions with people with AD and dementia variable was found to have the most associations with AD knowledge. It showed statistically significant association with risk factors (*F*(3,182) = 3.324, *p =* 0.021), symptoms (*F*(3,182) = 5.577, *p* = 0.001), assessment and diagnosis (*F*(3,182) = 3.926, *p* = 0.010), life impact (*F*(3,182) = 6.212, *p <* 0.001), caregiving (*F*(3,182) = 3.913, *p* = 0.010), and lastly for total ADKS score (*F*(3,182) = 6.832, *p* < 0.001). Table 4 outlines the univariate analyses for the domains.

**Table 4:** Univariate Analyses for Other Demographic Variables (*N* = 186)

Dependent Variables (ADKS domains)	Independent Variables					
	Gender *p*-value	Age p-value	Education Level p-value	Familiarity with AD/dementia p-value	Living with 65 or over people p-value	Interacted with AD/dementia p-value
Risk Factor	0.169	0.707	0.387	0.125	0.867	0.021*
Symptom	0.104	0.152	0.134	0.001**	0.788	0.001**
Course of the Disease	0.013*	0.055	0.096	0.089	0.300	0.065
Assessment and Diagnosis	0.733	0.708	0.259	0.143	0.939	0.010*
Treatment and Management	0.088	0.616	0.345	0.162	0.308	0.054
Life Impact	0.155	0.650	0.281	0.299	0.220	< 0.001**
Caregiving	0.149	0.095	0.497	0.177	< 0.001**	0.010*

*Significant at *p* < 0.05 **Significant at *p* < 0.01

Finally, MANOVA and MANCOVA tests were used for multivariate analysis across the domains for each independent variable. Using Wilk’s statistic, there was a significant effect of ethnicity on ADKS domains, Λ = 0.863, *F* (14,342) =1.875, *p* = 0.028, after controlling for gender, age, education, familiarity with dementia, living with those over 65, and interacting with those with dementia. There was also a significant effect on ADKS domains, for both living with those over 65, Λ = 0.844, *F* (14,354) =2.240, *p* = 0.006, and interacting with those with dementia, Λ = 0.735, *F* (21,506) =2.606, *p* < 0.001.

## Discussion

To the best of our knowledge, this study is the first to identify and compare knowledge and perceptions of AD amongst adults between 18 and 49 years of age of different ethnicities in the UK. The study had sufficient power to detect a medium effect size (0.25, *n* = 186) due to each group having at least 30 participants (80% power at a 5% significance level).

First, we investigated the AD knowledge of adults in the UK. We found that AD knowledge is not adequate among this 18–49 years old UK population regardless of ethnicity because total mean score was 13.5 out of 30 points. This finding confirms the few studies in the UK which show the AD knowledge is not adequate amongst a young population (from 18 to 43 years) [44, 46]. In our study, AD risk factors (35.3%) and symptoms (44.2%) knowledge were found to have a lower level of correct answers than course of the disease knowledge (Table 3). This contrasts with Hudson et al*’s* study of slightly older adults (mean age 42–43) in Britain, where both ‘risk factor’ and ‘course’ of AD had lower % correct answers than ‘life impact’ and ‘symptoms’ [44]. Importantly, a large proportion of those participants were unaware of risk factors that may increase one’s inclination to developing AD [44]. This indicates the importance of enhancing AD knowledge among this age group in UK, especially regarding AD risk factors. Similarly, knowledge of AD treatment has been found as poor amongst aged 18 and above people in 5 Europe countries (France, Germany, Italy, Spain, and UK) and dementia was not viewed as a health care priority [46]. In our study, only 47.3% of participants were able to correctly answer treatment and management related questions.

Second, we compared knowledge differences between three ethnic groups. We found that the primary knowledge differences between ethnicities were observed for the symptom area of AD although total ADKS score showed no association. In our study analysis revealed that White participants were significantly more likely to know about AD symptoms. This is in line with a previous study conducted in Florida, which showed significant differences for ethnicities in each domain of AD [17], although in an older population.

Third, we investigated potential demographic differences in AD knowledge and perceptions. Similarly to another study [41, 44, 49], we did not find a statistically significant association between ADKS scores and age. In contrast to our findings, other studies in various ages of populations have found that gender, and education level were positively correlated with individuals’ knowledge level of AD [26, 27, 39]. Our findings also revealed that interaction with people with AD or dementia had the most significant impact on ADKS domain’s knowledge scores amongst our participants. Likewise, previous studies, albeit in variable ages of population illustrated that interacting with people with dementia or AD was positively associated with people’s AD knowledge level [27, 41].

The abovementioned findings have several implications. With the increasing age of the UK population, there is much more need to determine the younger population’s AD knowledge and perceptions in order to define their knowledge needs. Determining knowledge needs is important especially in multicultural states because cultural differences affect individuals’ belief, perceptions and behaviours. This study adds to the existing body of literature, specific information regarding the knowledge perceptions amongst adults younger than 50 years of age of different ethnicities in the UK. Therefore, the lack of such studies in the UK has been mitigated by the study. However, in future research, the ethnic groups can be separated into their subgroups to provide more evidence to understand the knowledge differences of ethnicities and races. Further studies might also include other independent variables such as marital status and SES to provide comprehensive information for AD knowledge in the UK. Lastly, future studies might be conducted with larger sample group and wider ages of participants from the general population.

The study illustrated that adults younger than 50 years of age in UK have a low level of AD knowledge, supporting the need for the design and implementation of health communication interventions and policies to enhance AD knowledge. Meanwhile, the study not only examined total ADKS score, but also investigated ADKS domain score in order to specifically determine the respondents’ knowledge needs. The study identified the weakest areas of AD knowledge among participants, particularly in the content knowledge domain related to AD risk factors. Depending on the findings, it is recommended that health policymakers may provide the integrations of research, and clinical and social practice. The policymakers might also ensure evidence-based interventions and guidelines are provided that could maximise the AD knowledge level of populations in the UK. In order to overcome the low AD knowledge level, further studies can be conducted to define the causes of knowledge deficiencies. Furthermore, this study showed that ethnic groups differ in their knowledge and perceptions, especially between White and, Black or Asian participants regarding AD symptoms. As awareness is a prerequisite for changing behaviour (as shown in socio-cognitive models) [23–25], this study thus showed that (and how) health communication interventions need to target or tailor their health messages to different ethnic groups [24]. Thus, while designing health communication programs, health messages regarding AD symptoms need prioritisation when targeted communication is provided for Black or Asian individuals. Although the finding that interaction with those with dementia had the strongest association with several ADKS domains, it is perhaps to be expected, and has less implication for policy or interventions, although it highlights the importance of inclusion in demographics in future studies.

The study has several limitations. First, the cross-sectional study design was used in this research, so it could not establish causal relationship between the independent variables and the AD knowledge score. Low knowledge levels reasons such as language, stigmatisation, and economic factors, were not tested to compare amongst these ethnic groups in this study. Second, the study did not separate ethnic groups for their subgroups. For instance, it did not include Asian subgroups like Pakistani, Bangladeshi, or Indian. Third, it should be remarked that the study was a convenience sample of UK populations who were 18 or more years old, as it involved participants who had selected to be registered for the Prolific online recruitment panel and confirmed to be surveyed at the time of the Prolific invitation. Use of such a convenience sample might limit capability to generalize findings. Fourth, the study has only represented participants between 18 and 49 years old due to the study’s recruitment from those registered on an online recruitment panel which could have a bias for younger adults; therefore, our participants were not representing a wider age range representative of a general populations. Fifth, the chronic diseases associated with AD, like type-2 diabetes, hypertension, midlife obesity, having high cholesterol, depression, were not questioned to determine the AD developing risk for participants. Finally, to determine whether the participants’ information status was an obstacle in accessing the health system, it was not asked if the participants had difficulty accessing the health system. Thus, the findings cannot be interpreted as whether individuals do not access their health system because of their lack of AD knowledge. Hence, future research should be conducted with a more diverse and characterised sample.

## Conclusion

AD is a significant public health problem in the UK in a rapidly ageing population. Although most individuals with dementia or AD live in the community and are cared for by their family or friends, many studies show that AD knowledge is insufficient and there are knowledge and perception inequalities amongst different ethnic groups. Moreover, previous studies have mainly been inclined to investigate dementia or AD knowledge amongst older population (50 years or over) [17, 26, 27, 39, 41, 45]; hence, the study aimed to research (1) AD knowledge amongst adults between 18 and 49 years of age in the UK, (2) compare knowledge differences between ethnic groups, and (3) illustrate whether there were correlations between demographic characteristics and AD knowledge and perceptions . As a result, the study revealed that although there is not a big difference in knowledge of AD as examined with the ADKS between ethnic groups with a similar education, participants have inadequate AD knowledge, especially about AD risk factors. Additionally, Asian participants had lower ADKS score than White and Black people, while White individuals had significantly more knowledge about AD symptoms than Black participants. Lastly, interacting with people with dementia or AD were found to be positively correlated with individuals’ knowledge level of AD. The results indicate an urgent need for AD education programmes in the age group (younger than 50 years of age) in the UK, especially to those who do not have much interaction with those with AD or dementia, and the need to tailor health communication to ethnic groups based on different knowledge perceptions stipulated in this study.

## Data Availability

The datasets used and/or analysed during the current study are available from the corresponding author on reasonable request.

## References

[1] Martin Prince A, Wimo A, Guerchet M, Gemma-Claire Ali M, Wu Y-T, Prina M, et al. World Alzheimer Report 2015. The global impact of dementia: An analysis of prevalence, incidence, cost and trends 2015. www.alz.co.uk/worldreport2015corrections. Accessed 20 Jan 2021.

[2] PHE England. Dementia: applying All Our Health - GOV.UK. https://www.gov.uk/government/publications/dementia-applying-all-our-health/dementia-applying-all-our-health. Accessed 12 Oct 2020.

[3] Wittenberg R, Hu B, Barraza-Araiza L, Funder AR. CPEC working paper 5 the projections were produced using an updated version of a model developed by CPEC at LSE for the modelling outcome and cost impacts of interventions for dementia (MODEM) study. Disclaimer. 2019; www.modem-dementia.org.uk. Accessed 12 Aug 2020.

[4] Livingston G, Sommerlad A, Orgeta V, Costafreda SG, Huntley J, Ames D, Ballard C, Banerjee S, Burns A, Cohen-Mansfield J, Cooper C, Fox N, Gitlin LN, Howard R, Kales HC, Larson EB, Ritchie K, Rockwood K, Sampson EL, Samus Q, Schneider LS, Selbæk G, Teri L, Mukadam N (2017). The lancet commissions dementia prevention, intervention, and care. Lancet..

[5] Dening T, Sandilyan MB (2015). Dementia: definitions and types. Nurs Stand.

[6] 2019 Alzheimer’s disease facts and figures. Alzheimers Dement 2019;15:321–387. doi:10.1016/j.jalz.2019.01.010, 3.

[7] Georges J, Jansen S, Jackson J, Meyrieux A, Sadowska A, Selmes M (2008). Alzheimer’s disease in real life - the dementia carer’s survey. Int J Geriatr Psychiatry.

[8] Grøntvedt GR, Schröder TN, Sando SB, White L, Bråthen G, Doeller CF (2018). Alzheimer’s disease. Curr Biol.

[9] Vinson LD, Crowther MR, Austin AD, Ma M, Guin SM (2014). African Americans, mental health, and aging. Clin Gerontol.

[10] Dodel R, Belger M, Reed C, Wimo A, Jones RW, Happich M, Argimon JM, Bruno G, Vellas B, Haro JM (2015). Determinants of societal costs in Alzheimer’s disease: GERAS study baseline results. Alzheimers Dement.

[11] Jones RW, Romeo R, Trigg R, Knapp M, Sato A, King D, Niecko T, Lacey L, DADE Investigator Group (2015). Dependence in Alzheimer’s disease and service use costs, quality of life, and caregiver burden: the DADE study. Alzheimers Dement.

[12] Jönsson L, Lin P-J, Khachaturian AS (2017). Special topic section on health economics and public policy of Alzheimer’s disease. Alzheimers Dement.

[13] Tosto G, Bird TD, Bennett DA, Boeve BF, Brickman AM, Cruchaga C, Faber K, Foroud TM, Farlow M, Goate AM, Graff-Radford NR, Lantigua R, Manly J, Ottman R, Rosenberg R, Schaid DJ, Schupf N, Stern Y, Sweet RA, Mayeux R, for the National Institute on Aging Late-Onset Alzheimer Disease/National Cell Repository for Alzheimer Disease (NIA-LOAD/NCRAD) Family Study Group (2016). The role of cardiovascular risk factors and stroke in familial Alzheimer disease. JAMA Neurol.

[14] Kent BA, Mistlberger RE (2017). Sleep and hippocampal neurogenesis: implications for Alzheimer’s disease. Front Neuroendocrinol.

[15] Ulep MG, Saraon SK, McLea S (2018). Alzheimer Disease. J Nurse Pract.

[16] Lane CA, Hardy J, Schott JM (2018). Alzheimer’s disease. Eur J Neurol.

[17] Milani SA, Lloyd S, Cottler LB, Striley CW (2020). Racial and ethnic differences in Alzheimer’s disease knowledge among community-dwelling middle-aged and older adults in Florida. J Aging Health.

[18] Tappen RM, Gibson SE, Williams CL (2011). Explanations of AD in ethnic minority participants undergoing cognitive screening. Am J Alzheimers Dis Other Dement.

[19] Edwards GA, Gamez N, Escobedo G, Calderon O, Moreno-Gonzalez I. Modifiable risk factors for Alzheimer’s disease. Front Aging Neurosci. 2019. 10.3389/fnagi.2019.00146. 10.3389/fnagi.2019.00146 PMC6601685 31293412

[20] Mukadam N, Sommerlad A, Huntley J, Livingston G (2019). Population attributable fractions for risk factors for dementia in low-income and middle-income countries: an analysis using cross-sectional survey data. Lancet Glob Heal.

[21] Robert PH, Clairet S, Benoit M, Koutaich J, Bertogliati C, Tible O, Caci H, Borg M, Brocker P, Bedoucha P (2002). The apathy inventory: assessment of apathy and awareness in Alzheimer’s disease, Parkinson’s disease and mild cognitive impairment. Int J Geriatr Psychiatry..

[22] Morris R, Becker J. Cognitive neuropsychology of Alzheimer's disease. Oxford: Oxford University Press; 2004. https://books.google.com.tr/books?id=5r7DaH1KvbcC&dq=Cognitive+neuropsychology+of+Alzheimer%27s+disease&hl=tr&source=gbs_navlinks_s.

[23] Eldredge LKB (2016). Planning health promotion programs an intervention mapping approach.

[24] Cheung KL, Hors-Fraile S, de Vries H. How to use the integrated-change model to design digital health programs. In: Digital Health: Elsevier; 2021. p. 143–57.

[25] de Vries H. An integrated approach for understanding health behavior; the I-change model as an example. Psychol Behav Sci Int J. 2017;2(2). 10.19080/PBSIJ.2017.02.555585.

[26] Ayalon L, Areán PA (2004). Knowledge of Alzheimer’s disease in four ethnic groups of older adults. Int J Geriatr Psychiatry..

[27] Nielsen TR, Waldemar G (2016). Knowledge and perceptions of dementia and Alzheimer’s disease in four ethnic groups in Copenhagen, Denmark. Int J Geriatr Psychiatry.

[28] Gray HL, Jimenez DE, Cucciare MA, Tong HQ, Gallagher-Thompson D (2009). Ethnic differences in beliefs regarding alzheimer disease among dementia family caregivers. Am J Geriatr Psychiatry.

[29] Mukadam N, Cooper C, Livingston G (2011). A systematic review of ethnicity and pathways to care in dementia. Int J Geriatr Psychiatry..

[30] Cahill S, Pierce M, Werner P, Darley A, Bobersky A (2015). A systematic review of the Public’s knowledge and understanding of Alzheimer’s disease and dementia. Alzheimer Dis Assoc Disord.

[31] Parveen S, Peltier C, Oyebode JR (2017). Perceptions of dementia and use of services in minority ethnic communities: a scoping exercise. Health Soc Care Community.

[32] Casado BL, Hong M, Lee SE (2018). Attitudes toward Alzheimer’s care-seeking among Korean Americans: effects of knowledge, stigma, and subjective norm. Gerontologist..

[33] Mukadam N, Cooper C, Basit B, Livingston G (2011). Why do ethnic elders present later to UK dementia services? A qualitative study. Int Psychogeriatrics.

[34] Mukadam N, Lewis G, Mueller C, Werbeloff N, Stewart R, Livingston G (2019). Ethnic differences in cognition and age in people diagnosed with dementia: a study of electronic health records in two large mental healthcare providers. Int J Geriatr Psychiatry..

[35] Purandare N, Luthra V, Swarbrick C, Burns A (2007). Knowledge of dementia among south Asian (Indian) older people in Manchester, UK. Int J Geriatr Psychiatry.

[36] Nápoles AM, Chadiha L, Eversley R, Moreno-John G (2010). Developing culturally sensitive dementia caregiver interventions: are we there yet?. Am J Alzheimers Dis Other Dement.

[37] Sun F, Ong R, Burnette D (2012). The influence of ethnicity and culture on dementia caregiving. Am J Alzheimer’s Dis Other Dementiasr.

[38] Mahoney DF, Cloutterbuck J, Neary S, Zhan L (2005). African American, Chinese, and Latino family caregivers’ impressions of the onset and diagnosis of dementia: cross-cultural similarities and differences. Gerontologist..

[39] Leung AYM, Molassiotis A, Zhang J, Deng R, Liu M, Van IK (2020). Dementia literacy in the Greater Bay Area, China: Identifying the At-Risk Population and the Preferred Types of Mass Media for Receiving Dementia Information. Int J Environ Res Public Health.

[40] Scott Roberts J, Connell CM, Cisewski D, Hipps YG, Demissie S, Green RC. Differences Between African Americans and Whites in Their Perceptions of Alzheimer Disease. Alzheimer Dis Assoc Disord. 2003. 10.1097/00002093-200301000-00003 12621316

[41] Sun F, Gao X, Shen H, Burnette D (2014). Levels and correlates of knowledge about Alzheimer’s disease among older Chinese Americans. J Cross Cult Gerontol.

[42] Ezran C, Bonds MH, Miller AC, Cordier LF, Haruna J, Mwanawabenea D, Randriamanambintsoa M, Razanadrakato HTR, Ouenzar MA, Razafinjato BR, Murray M, Garchitorena A (2019). Assessing trends in the content of maternal and child care following a health system strengthening initiative in rural Madagascar: a longitudinal cohort study. PLoS Med.

[43] Amado DK, Brucki SMD (2018). Knowledge about alzheimer’s disease in the Brazilian population. Arq Neuropsiquiatr.

[44] Hudson JM, Pollux PMJ, Mistry B, Hobson S (2012). Beliefs about Alzheimer’s disease in Britain. Aging Ment Health.

[45] Ayalon L (2013). Re-examining ethnic differences in concerns, knowledge, and beliefs about Alzheimer’s disease: results from a national sample. Int J Geriatr Psychiatry..

[46] Jones RW, MacKell J, Berthet K, Knox S (2010). Assessing attitudes and behaviours surrounding Alzheimer’s disease in Europe: key findings of the important perspectives on Alzheimer’s care and treatment (IMPACT) survey. J Nutr Heal Aging.

[47] Etikan I (2016). Comparison of Convenience Sampling and Purposive Sampling. Am J Theor Appl Stat.

[48] Carpenter BD, Balsis S, Otilingam PG, Hanson PK, Gatz M (2009). The Alzheimer’s disease knowledge scale: development and psychometric properties. Gerontologist..

[49] Carpenter BD, Zoller SM, Balsis S, Otilingam PG, Gatz M (2011). Demographic and contextual factors related to knowledge about Alzheimer’s disease. Am J Alzheimers Dis Other Dement.

[50] Courtenay BC, Weidemann C (1985). The effects of a “don’t know” response on palmore’s facts on aging quizze. Gerontologist..

[51] El-Masry R, Elwasify M, Khafagi M (2018). Adaptation and Reliability of the Arabic Version of Alzheimer’s Disease Knowledge Scale (ADKS) among Sample of Middle aged and Elderly Egyptians Attending Outpatient Clinics in Mansoura University Hospital. Egypt J Community Med.

[52] Wang Y, Xiao LD, Luo Y, Xiao SY, Whitehead C, Davies O (2018). Community health professionals’ dementia knowledge, attitudes and care approach: a cross-sectional survey in Changsha, China. BMC Geriatr.

[53] Fundamental Statistics for Social Research: Step-by-step Calculations and ... - Duncan Cramer - Google Kitaplar. https://books.google.co.uk/books?hl=tr&lr=&id=LB7FrGHUqNoC&oi=fnd&pg=PR6&dq=cramer+D+1998&ots=cC3tTCAMrA&sig=4Vx3BAd0YOTMzlmmYJ4qDS2hFzE&redir_esc=y#v=onepage&q=cramer D 1998&f=false. Accessed 12 Aug 2020.

[54] The SAGE Dictionary of Statistics: A Practical Resource for Students in the ... - Duncan Cramer, Dennis Laurence Howitt - Google Kitaplar. https://books.google.co.uk/books?hl=tr&lr=&id=pa3_49Mpso4C&oi=fnd&pg=PP1&dq=cramer+D+and+Howitt+1998+SAGE+dictionary+for+statisitcs&ots=YrzghNf2Fw&sig=ULA1X0m-aM1razK7tLNOCasxVhY&redir_esc=y#v=onepage&q=cramer D and Howitt 1998 SAGE dictionary for statisitcs&f=false. Accessed 12 Aug 2020.

[55] Doane DP, Seward LE. Measuring skewness: a forgotten statistic? J Stat Educ. 2011;19(2). 10.1080/10691898.2011.11889611.

